# Antimicrobial evaluation of red, phytoalexin-rich sorghum food biocolorant

**DOI:** 10.1371/journal.pone.0194657

**Published:** 2018-03-21

**Authors:** Folachodé U. G. Akogou, Heidy M. W. den Besten, A. P. Polycarpe Kayodé, Vincenzo Fogliano, Anita R. Linnemann

**Affiliations:** 1 Laboratory of Valorization and Quality Management of Food Bio-Ingredients (LaBio), DNSA/FSA, Université d’Abomey-Calavi, Cotonou, Benin; 2 Food Quality and Design, Wageningen University, Wageningen, The Netherlands; 3 Laboratory of Food Microbiology, Wageningen University, Wageningen, The Netherlands; University of Campinas, BRAZIL

## Abstract

Sorghum (*Sorghum bicolor*) extract is traditionally used as red biocolorant in West Africa to colour foods, among which *wagashi*, a soft cheese. This biocolorant is a source of the phytoalexin apigeninidin and phenolic acids, and users claim that it has preservative effects next to its colouring properties. If such a claim can be scientifically substantiated, it adds a valuable functional property to this natural red colorant, thereby increasing its potential applications in the food industry. Hence, the present study evaluated the antimicrobial properties of dye sorghum extracts using challenge tests in broth and *wagashi* as a model of a popular food application. The alkaline extract and hot aqueous extract were used for dyeing *wagashi* by 87.7% and 12.3% of the traders, respectively. The dyeing procedure is perceived as a preservation strategy, and is also a means to maximise the revenues. However, results demonstrated that the application of sorghum biocolorant on *wagashi* had no inhibitory effect on the growth of fungi (*Penicillium chrysogenum*, *Cladosporium macrocarpum*) and *Escherichia coli* O157:H7. Furthermore, sorghum biocolorant in broth had no effect on growth of *Listeria monocytogenes* and *Escherichia coli* O157:H7. Consequently, the commonly used extracts for colouring soft West-African cheese did not show a preservative effect. In addition, dyeing did not affect the physico-chemical properties of *wagashi*. Still, the red colour hampered visual detection of microbial growth, thus clarifying the preservative effect reported by users.

## Introduction

Nowadays consumers prefer natural food colorants because of potential adverse effects of artificial colorants, like hyperactivity in children [1–3]. However, the use of natural colorants is more challenging since they are usually less stable and more sensitive to processing conditions such as low pH, high temperature and the characteristics of the food matrix in which they are applied [4]. This is especially an issue for industrial applications because of the requirement to have a guaranteed shelf life during which the colour does not change in intensity or shade [5].

Dye sorghum is a variety of sorghum (*Sorghum bicolor*) with characteristic dark red leaf sheaths, which is usually grown on the borders of crop fields in West Africa. These leaf sheaths are highly appreciated to extract a colouring agent as its application confers a stable bright red colour to foods [6,7]. Extracts from dye sorghum leaf sheaths contain significant amounts of 3-deoxyanthocyanidins, namely apigeninidin and luteolinidin, as well as phenolic acids, i.e. 4-hydroxybenzoic acid and *p*-coumaric acid [8]. Apigeninidin is a phenolic phytoalexin that plays a role in the defence mechanism of plants against pathogens and reportedly has preventive and antiproliferative properties against cancer [9–11]. Phenolic acids (like 4-hydroxybenzoic acid and *p*-coumaric acid) are common weak acids used as preservative in the food industry [12,13]. Thus, the natural extract from dye sorghum leaf sheaths holds promise for wider food and pharmaceutical applications [11,14–16].

Traditionally, dye sorghum extract has various applications in the West African region [8]. It is used to colour soft cheese, locally known as *wagashi*, and porridges, which are a major staple food for its consumers. *Wagashi* is a traditional unripened soft cheese produced in Benin and Nigeria from cow’s milk [17]. Basically, the preparation of *wagashi* involves separation of the curd from the whey by the proteolytic activity of a watery extract of cysteine peptidases (i.e. procerain, procerain B, CpCP-1, CpCP-2 and CpCP-3) from *Calotropis procera* [18–20]. *C*. *procera* is the plant commonly used to provide the coagulant for making this type of soft cheese [21,22]. The curd of the cheese contains all the fat, and three quarters of the protein from the whole milk [23].

*Wagashi* has a short shelf-life of about three days in the Sudanian and Sudano-Guinean climatic zones, where it is mostly produced in Benin [24,25]. Fungi (e.g. *Penicillium* and *Cladosporium* genera) and pathogens (e.g. *Escherichia coli* O157) contribute to spoilage and give rise to safety issues related to *wagashi* consumption [26]. Soaking in whey or dyeing with sorghum colorant are some endogenous practices used, presumably also to increase shelf life [21,23]. The inhibitory effect of apigeninidin on fungi and bacteria [27,28] suggests its potential as a food antimicrobial ingredient and as such could underpin the perception of users of its preservative effect. Nevertheless, data to substantiate the contribution of sorghum biocolorant to the presentation of African soft cheese are lacking. This study first evaluated the antimicrobial properties of the sorghum biocolorant in the laboratory, after which the current practices with respect to the production of *wagashi* in Benin were assessed.

## Materials and methods

### Extraction of sorghum biocolorant

Twenty five gram of dye sorghum leaf sheaths was weighed and washed with 0.5 L of demi water. Two litre of demi water was transferred into a bowl and heated to 90°C. The washed leaf sheaths were then added to the hot water and extracted for 10 min. Next, the mixture was allowed to cool, after which the watery extract was obtained by centrifugation at 5000×g during 15 min using a Heraeus instrument (Thermo Fisher Scientific, UK). The supernatant was filter-sterilised using a 0.2 μm AcroVac Filter Unit (VWR, Netherlands) to obtain a sterile cool watery extract of sorghum biocolorant.

### Determination of the anthocyanin composition

The apigeninidin, 4-hydroxybenzoic acid and *p-*coumaric acid contents of the sorghum biocolorant solutions were measured with an Ultimate 3000 RS High Performance Liquid Chromatography (HPLC) system equipped with a Diode Array Detector DAD-3000 RS (Thermo Scientific Dionex) and a quaternary pump LPG- 3000 RS (Thermo Scientific Dionex). The anthocyanins were separated with a Polaris C18-A column (150×4.6 mm, Varian) at a flow rate of 1 mL/min. The mobile phase consisted of formic acid (10%) in milli-Q water (A) and methanol (100%) (B). The total running time was 35 min and the elution gradient of B was planned as follows: 0 to 20 min, from 5% to 60% B; 20 to 25 min, from 60% to 100% B; 25 to 30 min with 100% B; 30 to 31 min from 100% to 5% B; 31 to 35 min with 5% B. The UV-spectra were recorded at 190–610 nm. The apigeninidin, 4-hydroxybenzoic acid and *p*-coumaric acid were measured at 480, 260 and 280 nm, respectively. Compounds were identified using the UV-spectra and the retention time of their corresponding standards. The following standards were used: apigeninidin (Extrasynthese, France), 4-hydroxybenzoic acid (Sigma Aldrich, Netherlands) and *p-*coumaric acid (Sigma Aldrich, Netherlands).

### *Wagashi* making in the laboratory

**Fig 1:**
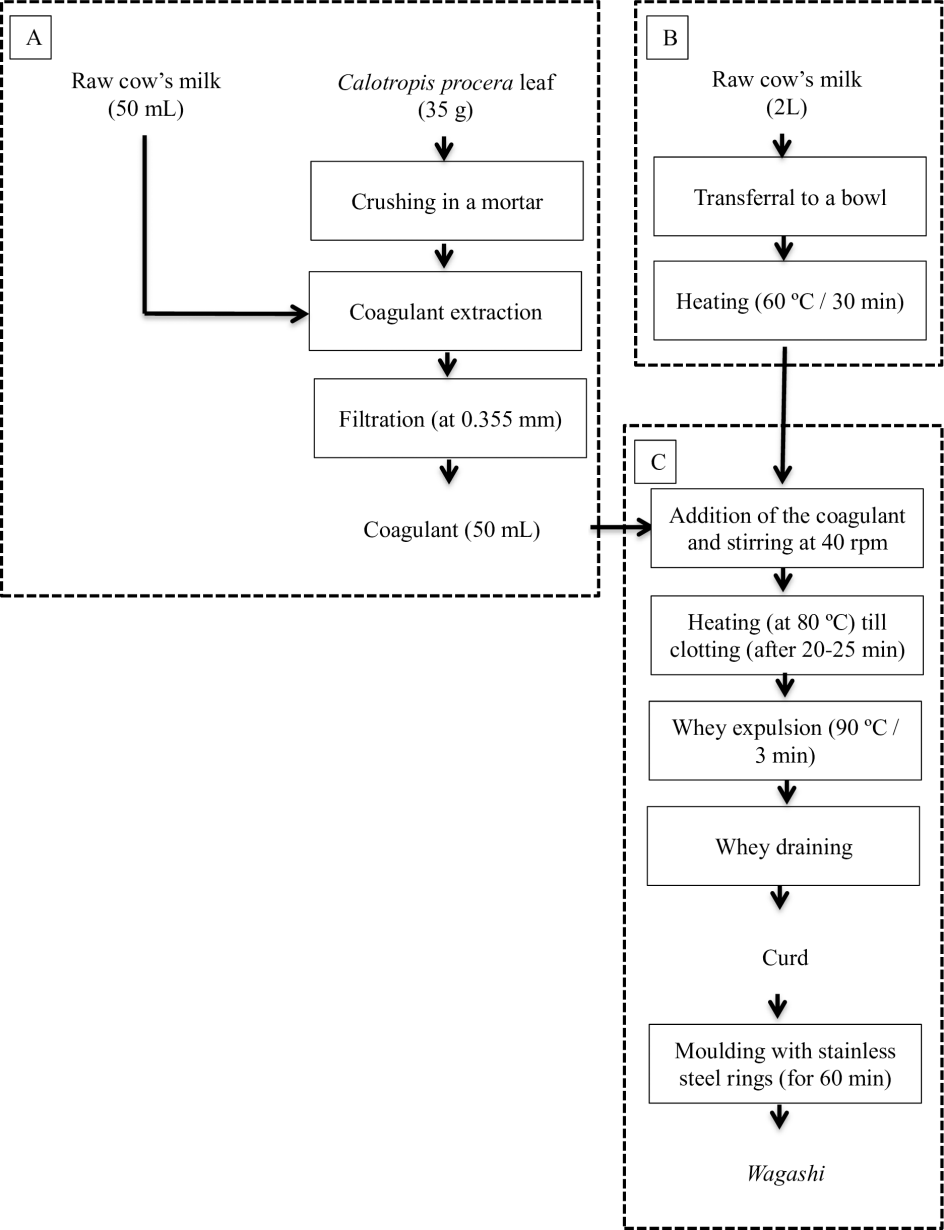
Flow chart of the coagulant extraction (A), the pre-treatment of the milk (B) and the clotting (C) in *wagashi* processing.

*Wagashi* was made using a thermomixer (Vorwerk, Germany) in accordance to the common cheese making practices in Benin (Fig 1). Two litres of fresh full milk were transferred into the bowl of the thermomixer and heated at 60°C for 30 min. Meanwhile, 35 g of *Calotropis procera* leaves, which were naturally grown in Benin and kept frozen at -20°C until needed, was crushed using a mortar. A volume of 50 mL of fresh milk was added to the crushed leaves to extract the coagulating cysteine peptidases. This extract was then filtered with a sieve (0.355 mm) to remove the *C*. *procera* residues, and added to the warm milk and stirred at 40 rpm. Next the temperature was raised to 80°C. When the clotting started after 20 to 25 min, the temperature was increased further to 90°C for 3 min to facilitate the expulsion of the whey. Next the curd was poured in sterilised stainless steel rings with a 44 mm inner-diameter and placed on a sterilised flame-tamer. The curd was left for one hour to allow the whey to drain. Finally the rings were removed, giving a cylindrical *wagashi* shape with a diameter of 44 mm and a height of 13 mm.

### Application of sorghum biocolorant on *wagashi*

The *wagashi* was dyed by soaking three cheese samples for 15 min either in sterile cool watery extract or in sterile hot watery extract of 60°C, obtained by warming sterile cool watery extract using a microwave (Panasonic 1670). Control samples were prepared by soaking cheese samples in demi water (negative control) or in a 0.1% solution of benzoic acid (positive control), based on the recommendation of the U.S Food and Drug Administration (FDA) to use benzoic acid as a food preservative at a maximum concentration of 0.1% [29]. The experiments for *wagashi* making and biocolorant extraction were performed in duplicate on different days. The S1 Fig. presents samples of non-dyed and dyed *wagashi*.

### Extraction of sorghum biocolorant for the challenge tests in broth

For the microbiological challenge tests in broth, concentrated extracts were used. These were prepared by adding 48 g of powder of dye sorghum leaf sheaths to either 0.5 L of demi water or 0.5 L of aqueous solution of *kanwu*, an alkaline rock salt that is often used in West Africa for food preparation, at 1.5 mg/mL. The extraction was performed at room temperature during 30 min under magnetic agitation. The watery extract of sorghum dye was collected by filtration using Whatman quantitative filter paper, ashless, Grade 42 (2.5μm) and was then filter-sterilized using a 0.2 μm AcroVac Filter Unit (VWR, Netherlands). The resulting sterile watery extracts were mixed in equal volumes with two times concentrated sterile Brain Heart Infusion (BHI) (Oxoid LTD, England) broth to obtain dyed BHI (50%). The control was prepared by mixing, again in equal volumes, two times concentrated sterile BHI broth and sterile demi-water.

### Inoculum preparation

All the microbial strains used were provided by the Laboratory of Food Microbiology (Wageningen University, Netherlands). The fungal strains (i.e. *Penicillium chrysogenum*, *Cladosporium macrocarpum*) were received in 1988 from the Centraalbureau voor Schimmelcultures (CBS, Utrecht, the Netherlands). Only the strain code of *Cladosporium macrocarpum* (i.e. 175.62) was available. One loop-full from a 3-day old culture of *Penicillium chrysogenum* as well as from a *Cladosporium macrocarpum* culture were inoculated on Malt Extract Agar slant tubes and incubated at 28°C (at 64% of humidity) for seven days. The spores were harvested by adding 9 mL of sterile peptone physiological salt solution (Tritium microbiologie, Netherlands) to the slant tubes and mixed with a vortex. The spore suspensions were collected and stored in cryovials with 30% of glycerol at -80°C. Cryovials were thawed at 4°C overnight before use. To determine the spore concentrations, a volume of 100 μL of the spore inoculum was transferred to 0.9 mL of peptone physiological salt solution (Tritium microbiologie, Netherlands). From this first dilution, series of 10-fold dilutions were made. From the dilutions, 100 μL of spore inoculum was spread on Dichloran-Glucose (DG 18) Agar Base (Oxoid LTD, England) plates and incubated at 25°C. The plate counts were determined after 24 and 48 h. The concentration of the inocula of *P*. *chrysogenum* and of *C*. *macrocarpum* were 6.3 log spores/mL and 5.6 log spores/mL, respectively. Serial decimal dilutions of the inocula in peptone physiological salt solution (Tritium microbiologie, Netherlands) were made to prepare 4.3 log spores/mL and 4.6 log spores/mL of *P*. *chrysogenum* and *C*. *macrocarpum*, respectively. These inocula and their dilutions were used for the challenge tests on *wagashi*.

Strains of *Escherichia coli* O157:H7 and *Listeria monocytogenes* were available in cryovials with 30% of glycerol at -80°C. Before use, they were streaked on BHI agar plates and incubated overnight at 30°C to obtain single colonies. One bacterial colony was transferred in BHI broth and incubated at 37°C for 24 h to obtain an inoculum of 9 log CFU/mL. Equal volumes of the inocula of five *E*. *coli* O157:H7 strains isolated from sheep (1), bovine (1), human (2) and a reference strain (ATCC 43895) were mixed to obtain a cocktail of *E*. *coli* O157:H7 to be used in the challenge tests on *wagashi*. Inocula of *E*. *coli* O157:H7 (from bovine origin) and *L*. *monocytogenes* (isolated from Mexican soft cheese) were also used for the growth tests in BHI broth.

### Microbiological challenge tests

#### Challenge test in broth

For the challenge tests in media, inocula of *E*. *coli* O157:H7 and *L*. *monocytogenes* were diluted in test tubes of BHI (dyed and plain) in order to achieve an initial concentration of 5 log CFU/mL. Inoculated tubes were incubated at 37°C. Samples were taken at 0, 1, 2, 4, 6, 8 and 24 h for monitoring bacterial growth. The bacterial counts were determined by making decimal dilutions in peptone physiological salt solution (Tritium microbiologie, Netherlands) and plating on BHI agar plates. The experiment was performed in duplicate and reproduced on a different day using freshly prepared materials.

#### Challenge test with *wagashi*

The challenge tests with *wagashi* were performed in a climate chamber (Weiss Enet environmental simulation, Weiss Technik) at a temperature of 28°C and relative humidity of 64%. These conditions correspond to those in the north of Benin [25], where *wagashi* is mostly produced. Samples of *wagashi* were surface-inoculated with 2 μL of 4.3 log spores/mL and 6.3 log spores/mL of *P*. *chrysogenum*, 2 μl of 4.6 log spores/mL and 5.6 log spores/mL of *C*. *macrocarpum* and 5 μL of 7.6 log CFU/mL of the *E*. *coli* O157:H7 cocktail. Previous studies investigating the quality and the safety of West African soft cheese, highlighted that *E*. *coli* and *L*. *monocytogenes* are the most relevant foodborne pathogens of this dairy product [26,30], with a lower prevalence for *L*. *monocytogenes* as compared to *E*. *coli* [31–33]. Therefore we focussed on *E*. *coli* 157:H7 in the application study on *wagashi*. The growing area on the *wagashi* was a circle with a diameter of 44 mm corresponding to a surface of 1519 mm^2^. Samples were incubated during 3 days. Three samples of *wagashi* inoculated with the *E*. *coli* cocktail were analysed at 0, 5, and 24 h, respectively. At 72 h, three samples inoculated with *P*. *chrysogenum* and *C*. *macrocarpum* were analysed. The growth of *E*. *coli* O157:H7 on *wagashi* was monitored by making a primary decimal dilution of each whole sample in peptone physiological salt solution (Tritium microbiologie, Netherlands) and homogenizing the sample using the stomacher, followed by a series of decimal dilutions in peptone physiological salt solution (Tritium microbiologie, Netherlands) and plate counting on Sorbital McConkey agar (Oxoid LTD, England). The growth of the fungal strains on *wagashi* was determined from pictures of the samples. The growth was assessed using ImageJ 1.46r, an open source Java-written program. The experiment was performed in triplicate and reproduced on a different day using freshly prepared *wagashi*, colorant extract, and freshly prepared microbial cultures.

### Physico-chemical parameters of *wagashi*

The pH, the dry matter and the acid value of *wagashi* were measured of two samples at 0 and 72 h for each replication. The pH of *wagashi* was measured using a pH meter (Phenomenal, Type pH100L, VWR). The dry matter was determined by drying samples at 100°C during 24 h with a drying oven (VENTI-Line VL 115, VWR). The acid value was determined using the AFNOR norms as described by Bup, Abi [34]. Briefly, a sample of *wagashi* (2 g) was weighted, crushed and dissolved in 50 mL of ethanol / diethyl ether (1/1). This mixture was homogenised during 5 min. Three drops of indicator (1% of phenolphthalein in ethanol) was added to the mixture and then titrated with 0.1 M KOH in ethanol until the end point of the indicator. The pH at the indicator end point in our samples was 9.5 ± 0.2. Data were expressed as mg KOH/g of sample dry matter (DM).

The acid value was calculated using the following formula (1):

$$Acid\;Value=\frac{56.1\times N\times V}{M}$$ (1)

in which

56.1: Molecular mass in g of KOH

N: Normality of KOH

V: Volume in ml of KOH

M: mass in g of sample in dry matter.

### Survey on the application of sorghum biocolorant on *wagashi*

A study was carried out to assess the traditional application methods of sorghum biocolorant and the perceptions associated to its use in three towns with a rich tradition in the production of dyed *wagashi* in Benin, Africa, i.e. Parakou (in Borgou department), Natitingou (in Atacora department) and in Dassa-Zoumè (in Collines department). In each town, 30 traders were interviewed. Data were collected using a questionnaire, which was administered in the local language after an one-day training. It contained questions about ethnicity, unit operations and ingredients used to produce dyed *wagashi*, the revenues generated from dyed and non-dyed *wagashi* and the reasons for dyeing *wagashi*. The increase in revenues (R_i_) due to dyeing of *wagashi* were calculated using formula (2). The respondents were selected from the main cheese market in each locality. The protocols used for data collection were approved by the Faculty of Agricultural Sciences of the University of Abomey-Calavi, Benin. Informed consent was obtained from all respondents.

$$R_{i}(\%)=\frac{R_{dyed}-R_{non-dyed}}{R_{non-dyed}}\times 100$$ (2)

Where:

R_i_ = increase in revenues

R_dyed_ = revenues from dyed *wagashi*

R_non-dyed_ = revenues from non-dyed *wagashi*

### Data analysis

The field survey data were compiled using Sphinx Plus^2^ v.4.5 (Le Sphinx Développement, Chavanod, France) software for survey management. The mean values of the areas of fungal growth, the bacterial counts, the dry matter, the pH and the acid values were calculated and the difference between dyed samples and the controls were evaluated by (a) one-way analysis (ANOVA) followed by Turkey’s post-hoc tests or (b) by Kruskal-Wallis and Mann-Whitney pair-wise tests if normality tests failed.

## Results

### Effects of sorghum biocolorant on the microbial and physico-chemical quality of *wagashi*

**Fig 2:**
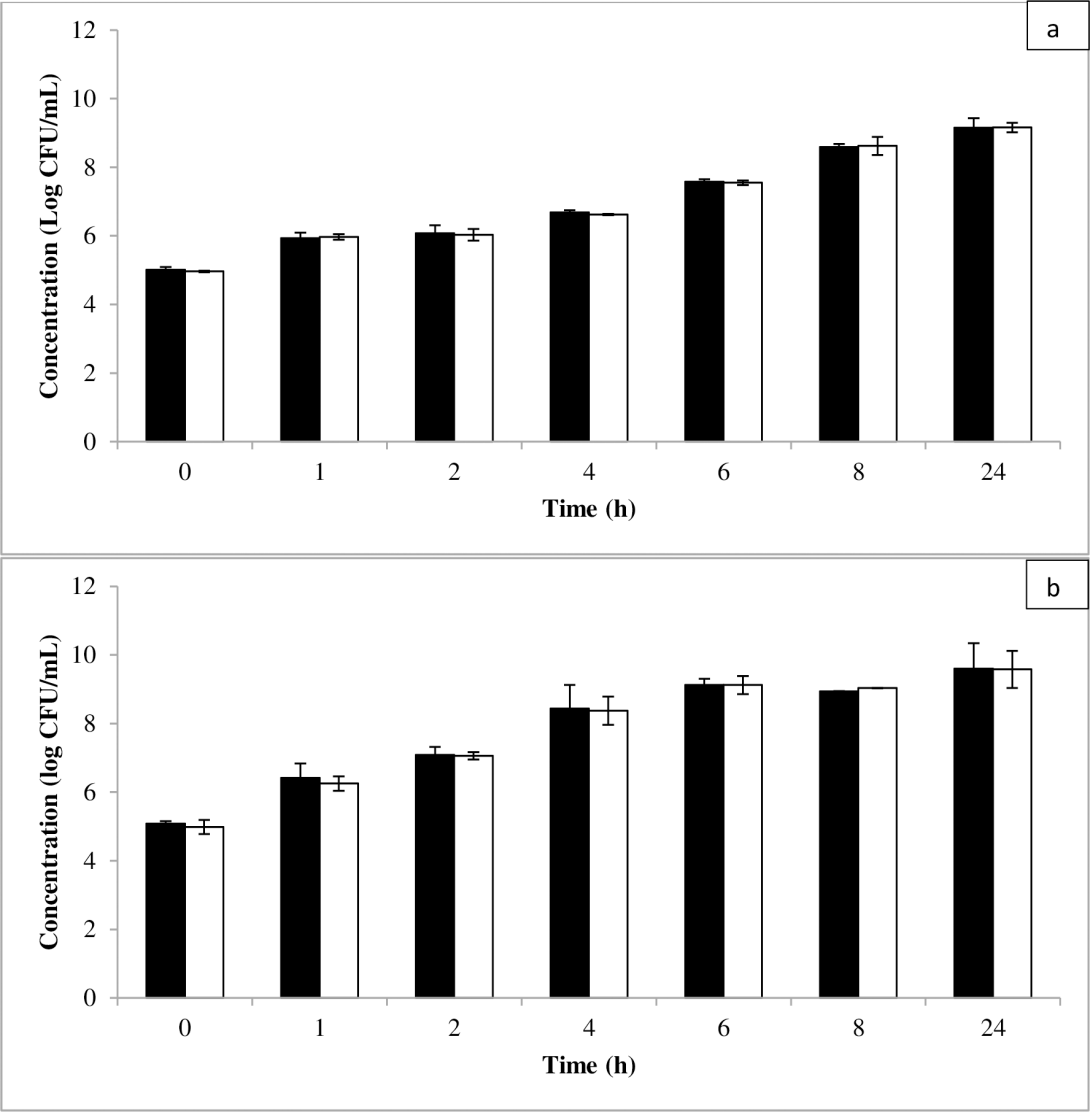
Growth of *Listeria monocytogenes* (a) and *Escherichia coli* O157:H7 (b) in dyed (■) and non-dyed (□) broth. Error bars represent standard deviations.

**Fig 3:**
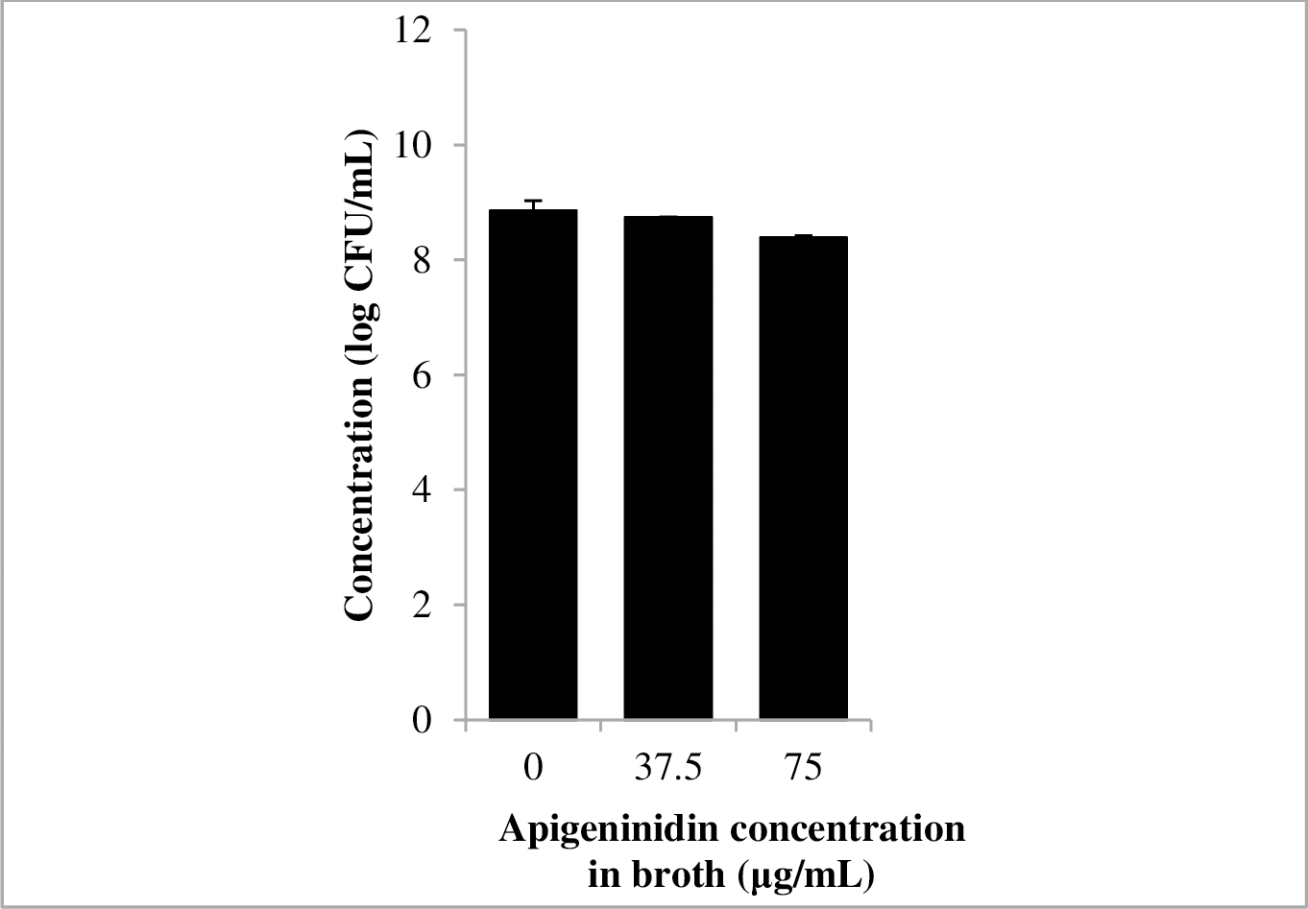
Growth of *Listeria monocytogenes* (■) in broth with increasing apigeninidin concentration after incubation for 24 h at 37°C (starting concentration 6.5 log CFU/mL). Error bars represent standard deviations.

Fig 2 presents the growth of *E*. *coli* O157:H7 and *L*. *monocytogenes* in dyed and non-dyed BHI. A dyed BHI with a final concentration of apigeninidin, 4-hydroxybenzoic acid and *p*-coumaric acid of 23.5, 6.8 and 3.95 μg/mL, respectively, did not affect the growth of pathogens (i.e. *L*. *monocytogenes* and *E*. *coli* O157:H7) in suspension (Fig 2A and 2B). Fig 3 presents the growth of *L*. *monocytogenes* at a 3.2 times higher concentration of apigeninidin. As most phenolic plant extracts usually have a less pronounced activity against Gram negative bacteria (e.g. *L*. *monocytogenes*) as compared to Gram positive bacteria (e.g. *E*. *coli*) [35,36], the test focused only on *L*. *monocytogenes*. The use of dyed BHI with a higher apigeninidin content (namely 75 μg/mL), as obtained by the extraction of sorghum biocolorant in a solution of *kanwu*, did still not affect the growth of *L*. *monocytogenes* in suspension (Fig 3).

**Fig 4:**
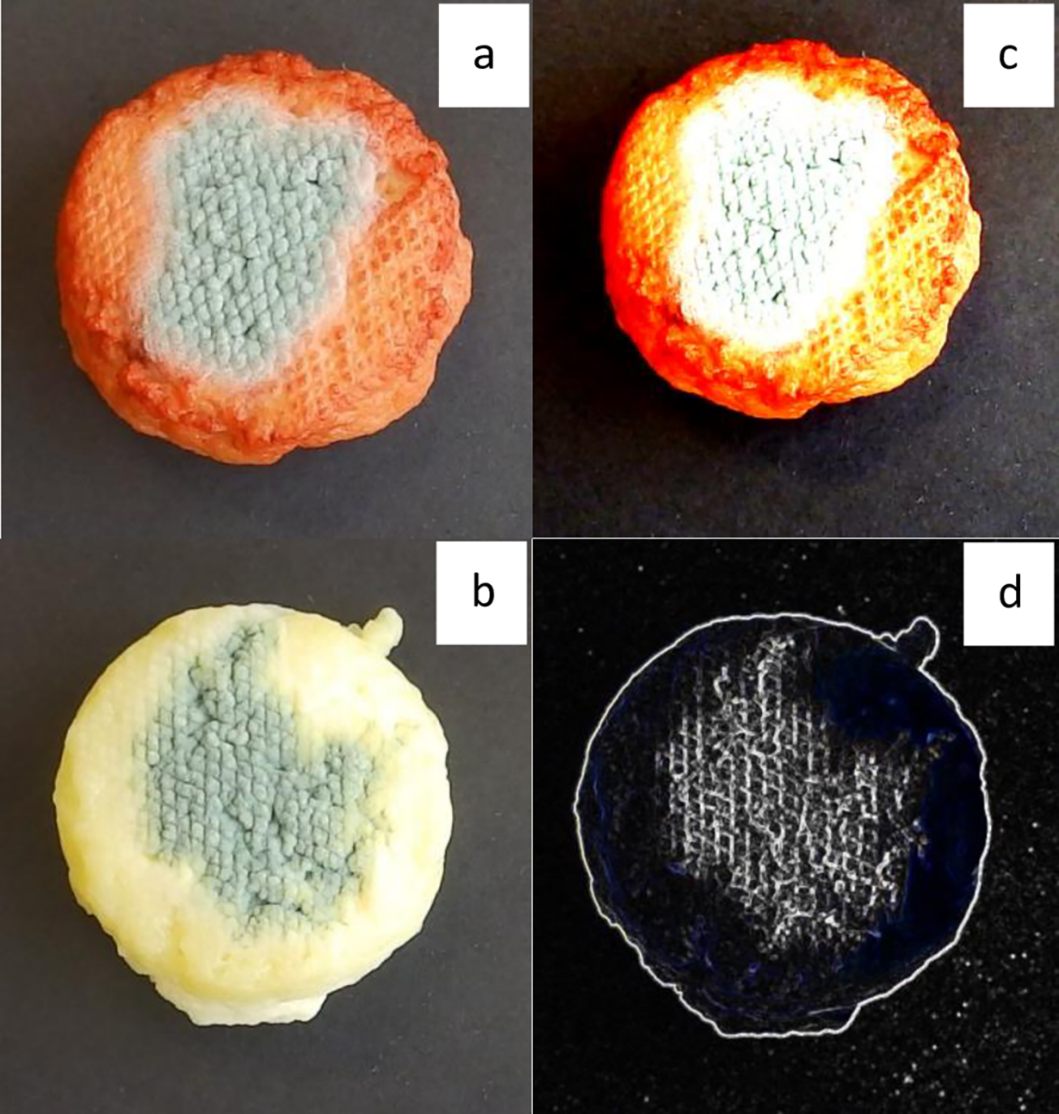
Analysis of the spreading area of fungi after growth on dyed (a) and control (b) *wagashi* with ImageJ 1.46r (c and d).

Dyeing *wagashi* using an alkaline extract would, next to the colouring effect, also increase the pH. Therefore, aqueous extracts were used to test the antimicrobial activity and potential physico-chemical changes. The growth of *P*. *chrysogenum* and *C*. *macrocarpum* was not inhibited on *wagashi* dyed with sorghum biocolorant (Table 1 and Fig 4). The sorghum biocolorant extract used for the test contained apigeninidin, 4-hydroxybenzoic acid and *p*-coumaric acid at 26.8, 3.3 and 2.3 μg/mL, respectively. In Table 1, two levels of contamination were tested for *P*. *chrysogenum* (i.e. 1.6 and 3.6 log spores) and for *C*. *macrocarpum* (i.e. 1.9 and 2.9 log spores). Apparently, fungi like *P*. *chrysogenum* and *C*. *macrocarpum* spread easily on dyed *wagashi*, even at a low spore contamination (Table 1). There was also no significant difference in the *E*. *coli* O157:H7 count on the dyed and the non-dyed *wagashi* samples (Table 2). This absence of an inhibitory effect indicates that the application of sorghum biocolorant, as done by traders in Benin, does not preserve *wagashi* from the proliferation of the fungi and pathogenic bacteria tested.

**Table 1:** Growth of fungi on *wagashi*.

		Spores spot-inoculated in the centre of *wagashi* (log spores)	Growth of fungi on *wagashi* (mm^2^) after storage at 28^o^ C for 72 h			
			Positive control (n = 6)	Negative control(n = 6)	Cool-dyed samples (n = 6)	Hot-dyed samples(n = 6)
***Penicillium chrysogenum***	1.6±0.0		1.0±0.1 a	1.0±0.4 a	2.0±1.0 a	1.8±0.7 a
	3.6±0.0		1.3± 0.4 a	1.0± 0.3 a	2.2± 0.1 b	2.5± 0.5 b
***Cladosporium macrocarpum***	1.9±0.0		0.80±0.1 a	0.7±0.2 a	0.6±0.2 a	0.6±0.1 a
	2.9±0.0		1.1±0.6 a	1.1±0.9 a	1.2±0.3 a	1.6±0.6 a

Mean ± Standard deviation; values with the same letter in the same row are not significantly different at 5%.

**Table 2:** Growth of *Escherichia coli* O157:H7 on *wagashi*.

Storage time(h)	*Escherichia coli* O157:H7 count (log CFU/g)	
	Control (n = 6)	Dyed *wagashi* (n = 6)
**0**	5.0±0.2 a	5.0±0.2 a
**5**	6.1±0.2 a	5.7±0.5 a
**24**	7.9±0.2 a	7.8±0.3 a

Mean ± Standard deviation; values with the same letter in the same row are not significantly different at 5%.

Although the application of natural biocolorant could have caused changes in the product properties of the soft cheese [37], the dyeing of *wagashi* with the aqueous extract did not affect the physico-chemical characteristics, namely the pH, dry matter, acid value, during the 3 days of storage (Table 3).

**Table 3:** Physicochemical characteristics of dyed *wagashi* over time.

	Storage time (h)	Control (n = 4)	Dyed *wagashi* (n = 4)
**pH**	0	6.7±0.1 a	6.7±0.1a
	72	6.4±0.9 a	6.5±0.4 a
**Acid value (mg KOH/g DM)**	0	4.9±2.7 a	4.2±2.1 a
	72	12.0±3.5 b	13.9±4.3 b
**Dry matter (%)**	0	31.1±3.0 a	32.8±2.7 a
	72	48.4±3.0 b	52.3±9.2 b

Mean ± Standard deviation; values with the same letter in the same row and sub-column are not significantly different at 5%.

### Survey on the application of sorghum biocolorant on *wagashi*

**Fig 5:**
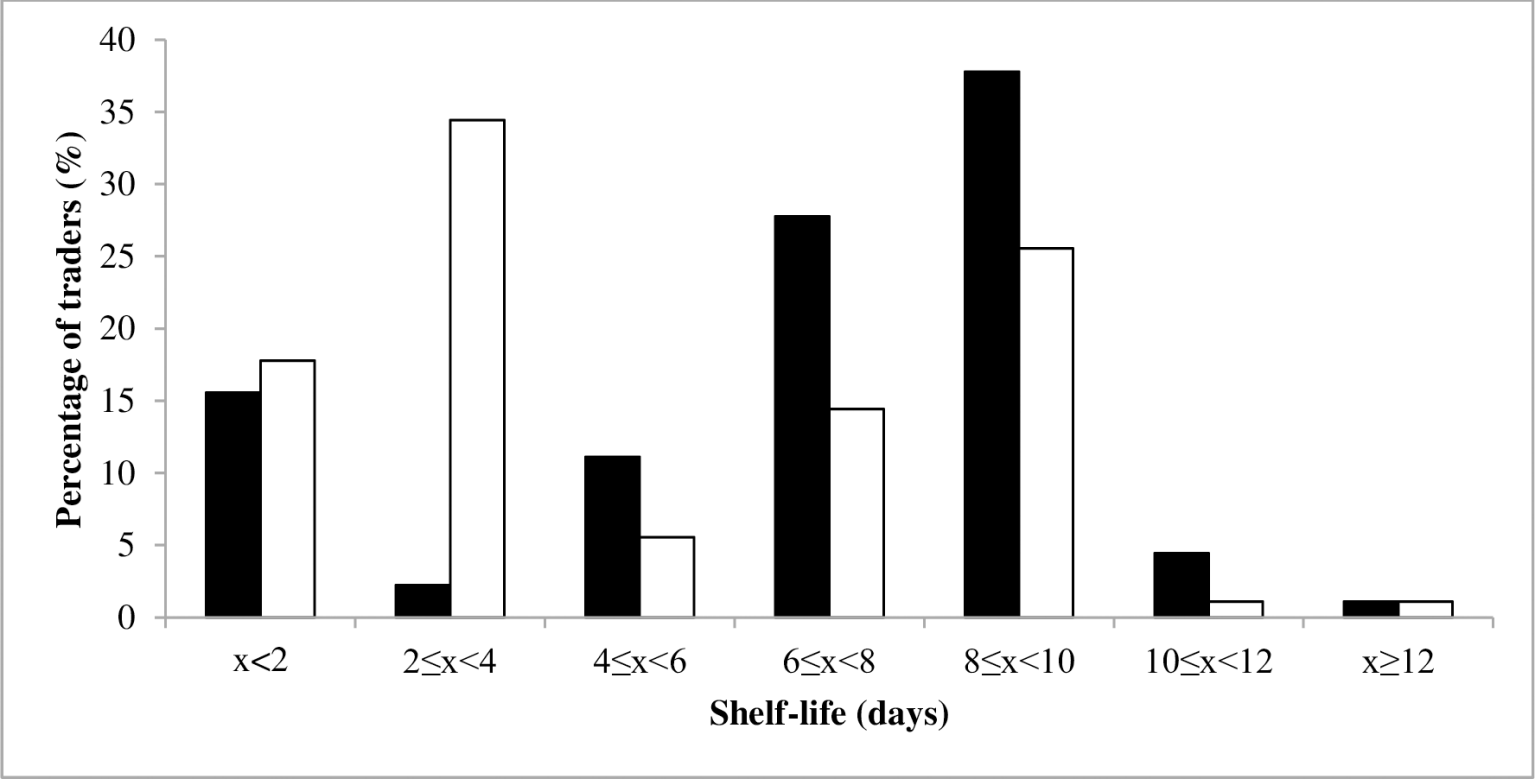
Percentage of traders willing to sell their *wagashi* when it is dyed (■) and non-dyed (□).

Traders usually buy non-dyed *wagashi* on a production site and transport it to the towns for sale after additional processing. The treatments traders apply to *wagashi* before selling the cheese are a heat treatment (97.8% of the traders) and dyeing (100% of the traders). As traders might have to keep *wagashi* several days at ambient temperature before sale, dyeing consists of a daily bath of *wagashi* in sorghum biocolorant and this practice is perceived as a way to prolong the product’s shelf-life. White *wagashi* is preferably sold before the fourth day or otherwise kept until the next market day, corresponding to a shelf life of 8–10 days (Fig 5). On the contrary, dyed *wagashi* may intentionally be kept for more than 8–10 days to speculate and maximize its revenues (Fig 5). Indeed, a mean revenue increase of 11.5% was recorded when *wagashi* was dyed.

Two methods are used to extract sorghum biocolorant, i.e. alkaline and hot aqueous extraction. Alkaline extraction is reported by 87.7% of the traders, although its use varies among traders. Indeed, *wagashi* could be cooked in the alkaline extract (24.4% of the traders) or let to soak in the cool alkaline extract (43.3% of the traders). Some traders (20%) suggest that the addition of warm water to the cool alkaline extract speeds up the dyeing process. The use of the hot aqueous extract (12.3% of the traders) is always associated to cooking of the extract with *wagashi*.

The dyeing of the *wagashi* by soaking it in an extract of sorghum biocolorant may last for up to two hours, although 52% of the traders apply a maximum soaking time of 30 min. The reasons to apply dye sorghum on *wagashi* are defined by traders as a way to enhance the sensorial quality (all traders), to keep the products looking fresh (mentioned by 49% of the traders), and to contribute to product preservation because its delays spoiling and increases the acidity (31% of the traders).

## Discussion

Bright colours are key factors in the perception of the sensorial quality of food products and therefore impact consumer choice and preference [38,39] Traders in Benin use the natural red sorghum colorant to make *wagashi* attractive for consumers. Adding a colouring agent to a food may affect consumers’ food preferences [40]. Hence, the colouring of *wagashi* could be considered as a value-adding unit operation by traders. The alkaline and hot aqueous extracts are commonly used extracts from dye sorghum to impact food colour in Benin [6]. These extracts differ in apigeninidin content, colour density and pH. The apigeninidin content and the colour density of the alkaline extract can be three times higher than that of the aqueous extract. In addition, the pH of the alkaline extract is around 8–9 whereas it is 7 for the hot aqueous extract [6]. An extract of 50 μM apigeninidin, equivalent to 13 μg/mL, was reported to inhibit the growth of fungi and bacteria (particularly Gram positive) [27,28]. However, the results of the challenge tests on *wagashi* and in broth with apigeninidin at levels of 23.5 μg/mL and 75 μg/mL, respectively, did not corroborate those findings. Although the antiproliferative activity of phenolic phytoalexins has been largely proven and is even proposed as a natural preservative to fungal pathogens [15,41–43], detoxification of phytoalexins by microorganisms could change their effectiveness as natural preservatives [44]. Naturally occurring weak acids, e.g. benzoic acid and *p*-coumaric, are widely used as food preservatives [12,45], and it has been reported that concentrations of 7 and 1.87 mg/mL of benzoic acid and *p*-coumaric, respectively, are required for an effective inhibition of pathogen growth in food matrices [12,46]. Conversely, the benzoic acid and *p*-coumaric acid contents in sorghum biocolorant apparently are not sufficient for preservative purposes. With total counts of 4.6 and 6.7 log CFU/g for *E*. *coli*, and moulds and yeasts, respectively, reported in samples of *wagashi* [26], use of sorghum biocolorant could not be acclaimed to function as a natural preservative in *wagashi*. As processors also use alkaline colorant extracts, it is recommended to also test the antimicrobial effects of these extracts in *wagashi*, though non-antibacterial activity was observed in the current study for the tested pathogens in broth. Although only 31% of traders clearly mention the use of sorghum extract as a food preservative in *wagashi*, this mistaken notion could present a risk for consumers when no heat treatment is applied to the cheese within 3 days of storage. Moreover, since microbial growth is better visible on *wagashi* that has not received a colouring treatment, it is possible that the red colorant masks microbial contamination, thus causing a food safety risk. Since the colorant is not sterilised, possible fungal and bacterial contaminants could be introduced via the leaf sheaths [47–49]. Washing of the sorghum leaf sheaths in potable water could be a first step to reduce microbial contaminants via the leaf sheaths [50]. Next, a hot extraction method at 86°C during 15 min could be recommended to inactivate most of the pathogenic cells [50,51].

The use of sorghum biocolorant could be of interest to the food industry, in particular the dairy and meat industries, looking for a stable natural colorant with a high colour density and potential health-supporting attributes (e.g. antioxidant properties) [6–8] [52–56]. However, this potential impact has to be investigated further.

## Conclusion

The anti-fungal and anti-bacterial properties of dye sorghum extracts could not be confirmed in nutrient-rich broth and on *wagashi*, a traditional West African soft cheese, as a model for a popular food application. Hence, it is likely that the most important attribute of the brightly red biocolorant from dye sorghum in Benin is its eye-catching property. This might open avenues for application as natural colorant in a variety of (dairy and meat-based) industrially produced foods, in particular when colour stability and potential health-supporting properties can be supported in further research.

## Supporting information

S1 Fig Non-dyed (A) and dyed (B) *wagashi* (with 44 mm diameter) used for the challenge tests.
(DOCX)

S1 Appendix Questionnaire des transformatrices de fromage local (*wagashi*). The original survey questionnaire in French as used in the study.
(DOCX)

S2 Appendix Questionnaire for processors of local soft cheese (*wagashi*). The survey questionnaire translated in English.
(DOCX)

## References

[1] Stevens LJ, Burgess JR, Stochelski MA, Kuczek T. Amounts of artificial food colors in commonly consumed beverages and potential behavioral implications for consumption in children. Clin Pediatr. 2014;53(2):133–140. 10.1177/0009922813502849 24037921

[2] Martins N, Roriz CL, Morales P, Barros L, Ferreira IC. Food colorants: challenges, opportunities and current desires of agro-industries to ensure consumer expectations and regulatory practices. Trends Food Sci Technol. 2016;52:1–15.

[3] McCann D, Barrett A, Cooper A, Crumpler D, Dalen L, Grimshaw K, et al Food additives and hyperactive behaviour in 3-year-old and 8/9-year-old children in the community: a randomised, double-blinded, placebo-controlled trial. The Lancet. 2007;370(9598):1560–1567. 10.1016/S0140-6736(07)61306-3 17825405

[4] Wrolstad RE, Culver CA. Alternatives to those artificial FD&C food colorants. Annu Rev Food Sci Technol. 2012;3:59–77. doi: 10.1146/annurev-food-022811-101118 22385164 10.1146/annurev-food-022811-101118

[5] Vegara S, Martí N, Mena P, Saura D, Valero M. Effect of pasteurization process and storage on color and shelf-life of pomegranate juices. LWT-Food Sci Technol. 2013;54(2):592–596.

[6] Akogou FUG, Kayodé APP, den Besten HMW, Linnemann AR. Extraction methods and food uses of a natural red colorant from dye sorghum. J Sci Food Agric. 2018;98(1):361–368. doi: 10.1002/jsfa.8479 28600852 10.1002/jsfa.8479

[7] Akogou FUG, Kayodé APP, den Besten HMW, Linnemann AR, Fogliano V. Effects of processing and storage on the stability of the red biocolorant apigeninidin from sorghum. LWT-Food Sci Technol. 2018;90:592–597.

[8] Kayodé AP, Nout MR, Linnemann AR, Hounhouigan JD, Berghofer E, Siebenhandl-Ehn S. Uncommonly high levels of 3-deoxyanthocyanidins and antioxidant capacity in the leaf sheaths of dye sorghum. J Agric Food Chem. 2011;59(4):1178–1184. doi: 10.1021/jf103963t 21322653 10.1021/jf103963t

[9] Shih C-H, Siu S-O, Ng R, Wong E, Chiu LC, Chu IK, et al Quantitative analysis of anticancer 3-deoxyanthocyanidins in infected sorghum seedlings. J Agric Food Chem. 2007;55(2):254–259. doi: 10.1021/jf062516t 17227050 10.1021/jf062516t

[10] Poloni A, Schirawski J. Red card for pathogens: phytoalexins in sorghum and maize. Molecules. 2014;19(7):9114–9133. doi: 10.3390/molecules19079114 24983861 10.3390/molecules19079114 PMC6271655

[11] Yang L, Browning JD, Awika JM. Sorghum 3-deoxyanthocyanins possess strong phase II enzyme inducer activity and cancer cell growth inhibition properties. J Agric Food Chem. 2009;57(5):1797–1804. doi: 10.1021/jf8035066 19256554 10.1021/jf8035066

[12] Stojković D, Petrović J, Soković M, Glamočlija J, Kukić-Marković J, Petrović S. In situ antioxidant and antimicrobial activities of naturally occurring caffeic acid, *p*-coumaric acid and rutin, using food systems. J Sci Food Agric. 2013;93(13):3205–3208. doi: 10.1002/jsfa.6156 23553578 10.1002/jsfa.6156

[13] Ling MP, Lien KW, Wu CH, Ni SP, Huang HY, Hsieh DP. Dietary exposure estimates for the food preservatives benzoic acid and sorbic acid in the total diet in Taiwan. J Agric Food Chem. 2015;63(7):2074–2082. doi: 10.1021/jf503987y 25633072 10.1021/jf503987y

[14] Petti C, Kushwaha R, Tateno M, Harman-Ware AE, Crocker M, Awika J, et al Mutagenesis breeding for increased 3-deoxyanthocyanidin accumulation in leaves of *Sorghum bicolor* (L.) Moench: a source of natural food pigment. J Agric Food Chem. 2014;62(6):1227–1232. doi: 10.1021/jf405324j 24460064 10.1021/jf405324j

[15] Kil HY, Seong ES, Ghimire BK, Chung I-M, Kwon SS, Goh EJ, et al Antioxidant and antimicrobial activities of crude sorghum extract. Food Chem. 2009;115(4):1234–1239.

[16] Sousa A, Araújo P, Azevedo J, Cruz L, Fernandes I, Mateus N, et al Antioxidant and antiproliferative properties of 3-deoxyanthocyanidins. Food Chem. 2016;192:142–148. doi: 10.1016/j.foodchem.2015.06.108 26304331 10.1016/j.foodchem.2015.06.108

[17] Aworh OC, Egounlety M. Preservation of West African soft cheese by chemical treatment. J Dairy Res. 1985;52(1):189–195.

[18] Dubey VK, Jagannadham M. Procerain, a stable cysteine protease from the latex of *Calotropis procera*. Phytochemistry. 2003;62(7):1057–1071. 12591258 10.1016/s0031-9422(02)00676-3

[19] Singh AN, Dubey VK. Exploring applications of procerain B, a novel protease from *Calotropis procera*, and characterization by N-terminal sequencing as well as peptide mass fingerprinting. Appl Biochem Biotechnol. 2011;164(5):573–580. doi: 10.1007/s12010-011-9158-6 21264687 10.1007/s12010-011-9158-6

[20] Ramos M, Araújo E, Jucá T, Monteiro-Moreira A, Vasconcelos I, Moreira R, et al New insights into the complex mixture of latex cysteine peptidases in *Calotropis procera*. Int J Biol Macromol. 2013;58:211–219. doi: 10.1016/j.ijbiomac.2013.04.001 23583491 10.1016/j.ijbiomac.2013.04.001

[21] Cadmus S, Ajunwa O, Adeleke A, Agada C, Awoyele A. Mini-review: theoretical and onsite evaluation of hazard potentials in the local production of wara; an indigenous West African soft cheese. Br Microbiol Res J. 2013;3(3):218–234.

[22] Raheem D, Suri N, Saris PE. The characterization and application of *Calotropis procera*, a coagulant in Nigerian wara cheese. Int J Food Sci Technol. 2007;42(2):220–223.

[23] Alalade O, Adeneye J. The effect of storage period on the chemical composition and coliform microflora of wara cheese. Int J Dairy Sci. 2006;1(2):126–130.

[24] Adetunji VO. Comparative assessment of the effect of crude extracts of *Carica papaya* and *Terminalia cattapa*, and a bacteriocin on vacuum-packed West African soft cheese (wara). Afr J Microbiol Res. 2008;2(10):272–276.

[25] Assogbadjo AE, Sinsin B, Codjia JTC, Van Damme P. Ecological diversity and pulp, seed and kernel production of the baobab (*Adansonia digitata*) in Benin. Belgian J Bot. 2005;138(1):47–56.

[26] Aïssi VM, Soumanou MM, Bankolè H, Toukourou F, de Souza CA. Evaluation of hygienic and mycological quality of local cheese marketed in Benin. Aust J Basic Appl Sci. 2009;3(3):2397–2404.

[27] Schutt C, Netzly D. Effect of apiforol and apigeninidin on growth of selected fungi. J Chem Ecol. 1991;17(11):2261–2266. doi: 10.1007/BF00988006 24258604 10.1007/BF00988006

[28] Stonecipher LL, Hurley PS, Netzly DH. Effect of apigeninidin on the growth of selected bacteria. J Chem Ecol. 1993;19(5):1021–1027. doi: 10.1007/BF00992535 24249081 10.1007/BF00992535

[29] FDA. Food additive status list: http://www.fda.gov/Food/IngredientsPackagingLabeling/FoodAdditivesIngredients/ucm091048.htm#ftnB; 2016 [May 17, 2016].

[30] Adetunji VO, Arigbede MI. Occurrence of E. coli O157: H7 and Listeria monocytogenes and identification of Hazard Analysis Critical Control Points (HACCPs) in production operations of a typical tropic cheese wara and yoghurt. Pak J Nutr. 2011.

[31] Adeyeye SAO, Adeyeye SAO. A preliminary study on the quality and safety of street-vended warankasi (a Nigerian soft white cheese) from Ibadan, Oyo state, Nigeria. Br Food J. 2017;119(2):322–330.

[32] Saliu BK, Agbabiaka TO, Sule IO, Gambari-Ambali RO. Assessment of local methods of processing for the preservation of the physico-chemical properties and microbiological quality of two local cheeses in Ilorin, Nigeria. J Microbiol Biotechnol and Food Sci. 2014;3(4):337.

[33] Omemu A, Taiwo G, Obuotor T. Microbiological assessment and prevalence of food borne pathogens in street vended Wara-Nigerian white cheese. Am J Food Nutr. 2014;2(4):59–62.

[34] Bup DN, Abi CF, Tenin D, Kapseu C, Tchiegang C. Optimisation of the cooking process of sheanut kernels (*Vitellaria paradoxa* Gaertn.) using the Doehlert experimental design. Food Bioprocess Technol. 2012;5(1):108–117.

[35] Nostro A, Germano M, D’angelo V, Marino A, Cannatelli M. Extraction methods and bioautography for evaluation of medicinal plant antimicrobial activity. Lett Appl Microbiol. 2000;30(5):379–384. 10792667 10.1046/j.1472-765x.2000.00731.x

[36] Butkhup L, Chowtivannakul S, Gaensakoo R, Prathepha P, Samappito S. Study of the phenolic composition of Shiraz red grape cultivar (Vitis vinifera L.) cultivated in north-eastern Thailand and its antioxidant and antimicrobial activity. S Afr J Enol Vitic. 2016;31(2):89–98.

[37] Han J, Britten M, St-Gelais D, Champagne CP, Fustier P, Salmieri S, et al Effect of polyphenolic ingredients on physical characteristics of cheese. Food Res Int. 2011;44(1):494–497.

[38] Koch C, Koch EC. Preconceptions of taste based on color. J Psychol. 2003;137(3):233–242. doi: 10.1080/00223980309600611 12795546 10.1080/00223980309600611

[39] Resurreccion A. Sensory aspects of consumer choices for meat and meat products. Meat Sci. 2004;66(1):11–20. doi: 10.1016/S0309-1740(03)00021-4 22063927 10.1016/S0309-1740(03)00021-4

[40] Clydesdale FM. Color as a factor in food choice. Crit Rev Food Sci Nutr. 1993;33(1):83–101. doi: 10.1080/10408399309527614 8424857 10.1080/10408399309527614

[41] Sanzani SM, Schena L, Ippolito A. Effectiveness of phenolic compounds against citrus green mould. Molecules. 2014;19(8):12500–12508. doi: 10.3390/molecules190812500 25153867 10.3390/molecules190812500 PMC6270851

[42] Klančnik A, Guzej B, Hadolin Kolar M, Abramovič H, Smole Možina S. In vitro antimicrobial and antioxidant activity of commercial rosemary extract formulations. J Food Prot. 2009;72(8):1744–1752. 19722413 10.4315/0362-028x-72.8.1744

[43] Hasegawa M, Mitsuhara I, Seo S, Okada K, Yamane H, Iwai T, et al Analysis on blast fungus-responsive characters of a flavonoid phytoalexin sakuranetin; accumulation in infected rice leaves, antifungal activity and detoxification by fungus. Molecules. 2014;19(8):11404–11418. doi: 10.3390/molecules190811404 25093982 10.3390/molecules190811404 PMC6271790

[44] Jeandet P. Phytoalexins: current progress and future prospects. Molecules. 2015;20(2):2770–2774.

[45] Brul S, Coote P. Preservative agents in foods: mode of action and microbial resistance mechanisms. Int J Food Microbiol. 1999;50(1):1–17. 10.1016/s0168-1605(99)00072-0 10488839

[46] Cruz-Romero M, Murphy T, Morris M, Cummins E, Kerry J. Antimicrobial activity of chitosan, organic acids and nano-sized solubilisates for potential use in smart antimicrobially-active packaging for potential food applications. Food Control. 2013;34(2):393–397.

[47] Gadgil A. Drinking water in developing countries. Annu Rev Energ Environ. 1998;23(1):253–286.

[48] Johnson RC, Segla H, Dougnon TV, Boni G, Bankole HS, Houssou C, et al Situation of water, hygiene and sanitation in a peri-urban area in Benin, West Africa: the case of Sèmè-Podji. J Environ Prot. 2014;5(12):1277–1283.

[49] Dekker DM, Krumkamp R, Sarpong N, Frickmann H, Boahen KG, Frimpong M, et al Drinking water from dug wells in rural Ghana—*Salmonella* contamination, environmental factors, and genotypes. Int J Environ Res Public Health. 2015;12(4):3535–3546. doi: 10.3390/ijerph120403535 25826395 10.3390/ijerph120403535 PMC4410201

[50] Ameyapoh Y, de Souza C, Traore AS. Hygienic quality of traditional processing and stability of tomato (*Lycopersicon esculentum*) puree in Togo. Bioresour Technol. 2008;99(13):5798–5803. doi: 10.1016/j.biortech.2007.10.035 18164617 10.1016/j.biortech.2007.10.035

[51] Nazarowec-White M, Farber J. Thermal resistance of *Enterobacter sakazakii* in reconstituted dried‐infant formula. Lett Appl Microbiol. 1997;24(1):9–13. 9023998 10.1046/j.1472-765x.1997.00328.x

[52] Kumar H, Salminen S, Verhagen H, Rowland I, Heimbach J, Bañares S, et al Novel probiotics and prebiotics: road to the market. Curr Opin Biotechnol. 2015;32:99–103. doi: 10.1016/j.copbio.2014.11.021 25499742 10.1016/j.copbio.2014.11.021

[53] Hidalgo M, Oruna-Concha MJ, Kolida S, Walton GE, Kallithraka S, Spencer JP, et al Metabolism of anthocyanins by human gut microflora and their influence on gut bacterial growth. J Agric Food Chem. 2012;60(15):3882–3890. doi: 10.1021/jf3002153 22439618 10.1021/jf3002153

[54] Boto-Ordóñez M, Urpi-Sarda M, Queipo-Ortuño MI, Tulipani S, Tinahones FJ, Andres-Lacueva C. High levels of Bifidobacteria are associated with increased levels of anthocyanin microbial metabolites: a randomized clinical trial. Food Funct. 2014;5(8):1932–1938. doi: 10.1039/c4fo00029c 24958563 10.1039/c4fo00029c

[55] Gallego CG, Salminen S. Novel probiotics and prebiotics: how can they help in human gut microbiota dysbiosis? Appl Food Biotechnol. 2016;3(2):72–81.

[56] Kumar Y, Yadav DN, Ahmad T, Narsaiah K. Recent trends in the use of natural antioxidants for meat and meat products. Compr Rev Food Sci Food Saf. 2015;14(6):796–812.

