# Supplementary figures and images for: Antimicrobial evaluation of red, phytoalexin-rich sorghum food biocolorant
Source: PLoS One. 2018 Mar 21;13(3):e0194657. doi: 10.1371/journal.pone.0194657 (PMC5862489; doi:10.1371/journal.pone.0194657)

B

A


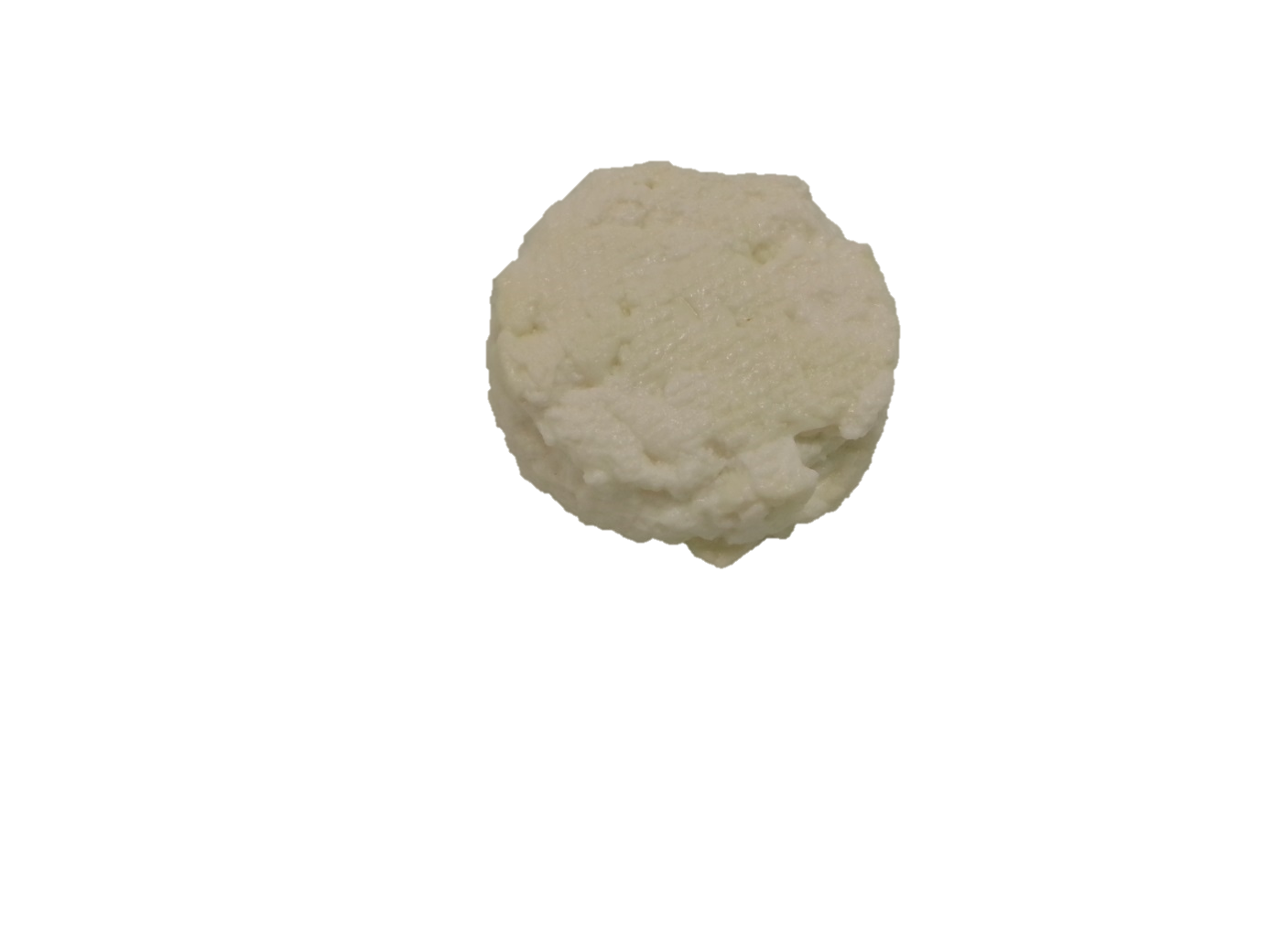

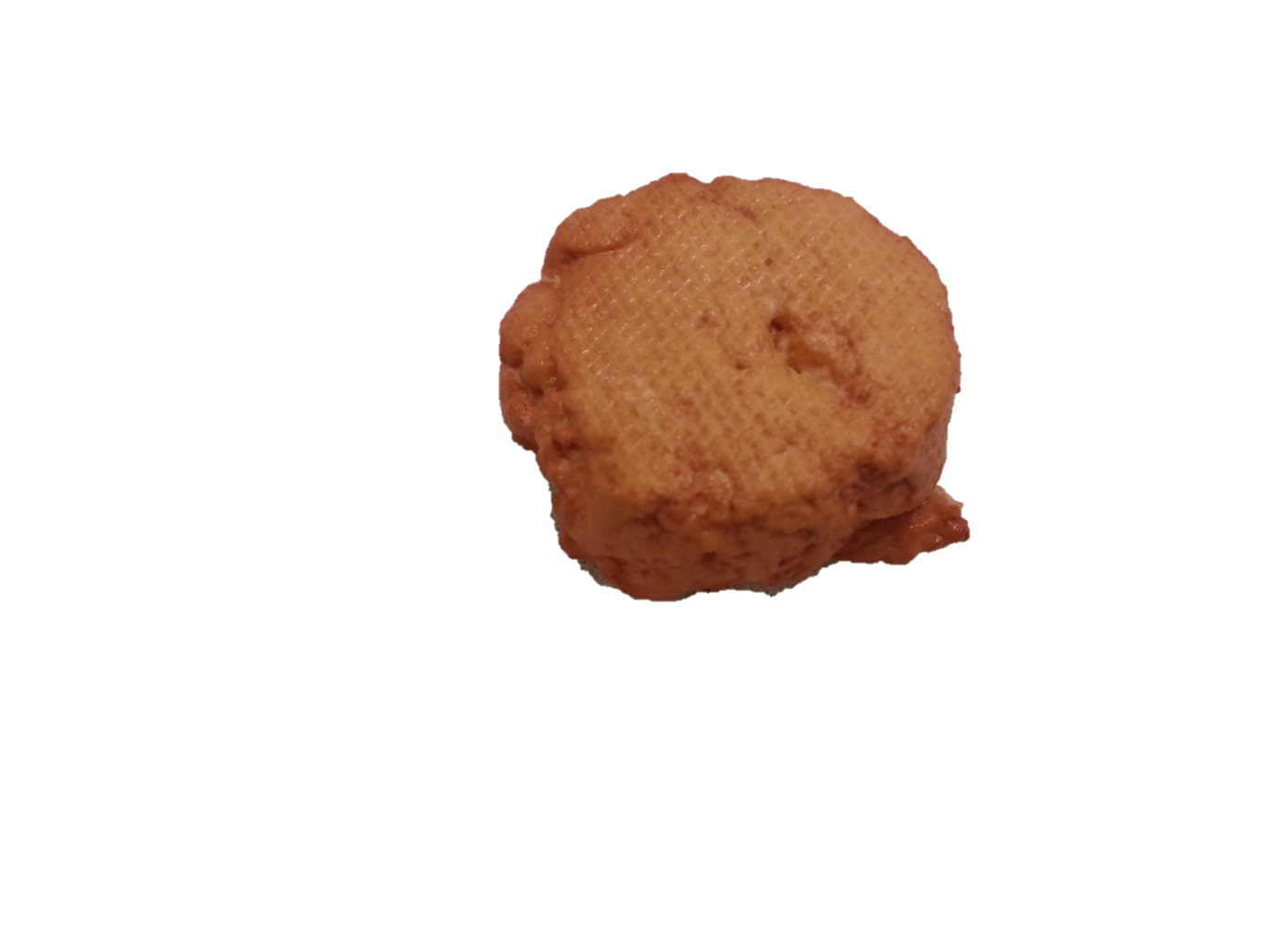


**S1 Fig. Non-dyed (A) and dyed (B) *wagashi* (with 44 mm diameter) used for the challenge tests**

Supplement: S1 Fig — Non-dyed (A) and dyed (B) wagashi (with 44 mm diameter) used for the challenge tests. (DOCX) [file pone.0194657.s001.docx]
