# Supplementary material for: Antimicrobial evaluation of red, phytoalexin-rich sorghum food biocolorant
Source: PLoS One. 2018 Mar 21;13(3):e0194657. doi: 10.1371/journal.pone.0194657 (PMC5862489; doi:10.1371/journal.pone.0194657)
Supplement: S1 Appendix — The original survey questionnaire in French as used in the study. (DOCX) [file pone.0194657.s002.docx]

**S1 Appendix. Questionnaire des transformatrices de fromage local (*wagashi*)**

(Questionnaire individuel destiné aux transformatrices de fromage local utilisant la gaine de sorgho colorant)

Fiche N° : …………. Date de l’enquête :……/………/………

Nom de l’enquêteur : ……………………………………………………

Généralités

| Caractéristiques | Modalités (à pré-remplir avant l’enquête) |
| --- | --- |
| Département |  |
| Commune |  |
| Arrondissement |  |
| Village |  |
| Hameau ou quartier |  |

**Identification de l’enquêté**

1-Nom :………………………………………………………………….

2-Prénom :………………………………………………………………………

3-Age :………… 4-Sexe : masculin féminin

5-Ethnie : bariba ;peuhl ;somba ;otamari ;natimba ;pila-pila lokpa ;yandé ;berba ;lama ;mahi ;nago autres (à préciser)……………………………………………………………..

6-Religion : musulman ; chrétien ; religion traditionnelle sans religion Autres religions (à préciser) ……………………………….

7-Niveau d’éducation : non lettré lettré niveau primaire

lettré niveau secondaire lettré niveau universitaire

9-Profession/occupation :

1. Comment colorez-vous le *wagashi* ?

| Opérations unitaires | Réponse | | Ingrédients | Réponse | |
| --- | --- | --- | --- | --- | --- |
|  | Oui | Non |  | Oui | Non |
| Cuisson du *wagashi* |  |  | *Kanmou* ou *kanwanboguo* |  |  |
|  |  |  | Bicarbonate |  |  |
|  |  |  | Sel |  |  |
| Extraction du colorant :  à froid à chaud | | | *Kanmou* |  |  |
|  |  |  | Cendres |  |  |
|  |  |  | Bicarbonate |  |  |

| Opérations unitaires | Durée de l’opération unitaire | Réponse | |
| --- | --- | --- | --- |
|  |  | Oui | Non |
| Coloration du *wagashi*:  dans de l’eau à température ambiante  dans de l’eau tiède  au cours du chauffage du *wagashi* | Moins de 15 minutes |  |  |
|  | 15 minutes à 30 minutes |  |  |
|  | 30 minutes à 45 minutes |  |  |
|  | 45 minutes à 1 heure |  |  |
|  | 1 heure à 1 heure 30 minutes |  |  |
|  | 1 heure 30 minutes à 2 heures |  |  |
|  | Autres durée (à préciser) |  |  |

1. Est-ce qu’un colorant de couleur plus intense permet de réduire la durée de la coloration de *wagashi* ?

Oui Non

1. Quelles sont les raisons de la coloration du *wagashi* ?

| Raisons | Oui | Non |
| --- | --- | --- |
| Rendre le *wagashi* attrayant |  |  |
| Masquer les tâches du *wagashi*  et uniformiser la couleur |  |  |
| Retarde la pourriture du *wagashi* |  |  |
| Retarde la fermentation du produit |  |  |
| Autres : (à préciser) |  |  |

1. Quelle est la durée de conservation des *wagashi* suivant ?

|  | Indication de la durée de conservation |
| --- | --- |
| *Wagashi* blanc acheté directement chez les peulhs | Faible moyenne longue |
| *Wagashi* cuit avec sel et *kanmou* | Faible moyenne longue |
| *Wagashi* cuit avec sel et *kanmou* et coloré en rouge avec les gaines de sorgho colorant | Faible moyenne longue |

1. En période de mévente quelle est la durée de conservation maximale que vous avez déjà enregistrée pour le *wagashi*rouge?

|  | Durée de conservation maximale |
| --- | --- |
|  |  |
| *Wagashi* rouge | 1 jour 2 jours 3 jours  4 jours 5jours 6 jours  7 jours 8 jours 9 jours  10 jours 11 jours 12 jours  Plus de 12 jours |

1. Et si c’était du *wagashi* blanc pourrait on atteindre une telle durée de conservation ?

|  | Durée de conservation maximale |
| --- | --- |
|  |  |
| *Wagashi* blanc | 1 jour 2 jours 3 jours  4 jours 5 jours 6 jours  7 jours 8 jours 9 jours  10 jours 11 jours 12 jours  Plus de 12 jours |

1. En cas de mévente, préciser la fréquence de recuisson et de recoloration du *wagashi*.

|  | Fréquence de nouvelles cuissons et de recolorations |
| --- | --- |
|  |  |
| Nouvelle cuisson avec du sel et *kanmou* | 1 jour 2 jours 3 jours  4 jours 5 jours 6 jours |
| Recoloration | 1 jour 2 jours 3 jours  4 jours 5 jours 6 jours |

1. Quelle taille de *wagashi* vendez-vous ?

Petite taille Taille moyenne Grande taille

1. Pourquoi avez-vous choisi les *wagashi* de cette taille ?

………………………………………………………………………………………………………………………………………………………………………………………………………………………………………………………………………………………………………

1. Est-ce que la quantité de gaines utilisée est différente selon la taille du *wagashi*?

Oui Non

Si oui expliquez votre réponse :……………………………………………………....................

…………………………………………………………………………………………………...

…………………………………………………………………………………………………...

…………………………………………………………………………………………………..

1. Est-ce que la durée de séjour dans l’eau de coloration est différente selon la taille du *wagashi*?

Oui Non

Si oui expliquez votre réponse :……………………………………………………....................

…………………………………………………………………………………………………...

…………………………………………………………………………………………………...

…………………………………………………………………………………………………..

1. Veuillez préciser les coûts des matières utilisées

| Etapes | Matières premières | Quantité | Prix | Coût |
| --- | --- | --- | --- | --- |
| Cuisson du *wagashi* | *Wagashi* |  |  |  |
|  | Eau |  |  |  |
|  | *Kanmou* |  |  |  |
|  | Sel |  |  |  |
|  | Sachets |  |  |  |
|  | Bois/Charbon |  |  |  |
|  | Autres |  |  |  |
|  | Total 1 |  |  |  |
| Coloration du *wagashi* | Gaines de sorgho (Nombre de botte) |  |  |  |
|  | Eau |  |  |  |
|  | *Kanmou* /Cendres |  |  |  |
|  | Autres |  |  |  |
|  | Total 2 |  |  |  |

1. Quel est le prix de votre *wagashi* rouge ?

………………………………………………………………………………………………..

1. Quel est le prix de votre *wagashi* blanc sans la coloration?

………………………………………………………………………………………………..

1. Quel est le temps alloué à chaque tâche ?

| Tâche | Temps alloué (minutes) |
| --- | --- |
| Cuisson du *wagashi* |  |
| Coloration du *wagashi* |  |
| Total |  |
