# Supplementary material for: Antimicrobial evaluation of red, phytoalexin-rich sorghum food biocolorant
Source: PLoS One. 2018 Mar 21;13(3):e0194657. doi: 10.1371/journal.pone.0194657 (PMC5862489; doi:10.1371/journal.pone.0194657)
Supplement: S2 Appendix — The survey questionnaire translated in English. (DOCX) [file pone.0194657.s003.docx]

**S2 Appendix. Questionnaire for processors of local soft cheese (*wagashi*)**

Sheet N° : …………. Date of the interview :……/………/………

Name of the investigator : ……………………………………………………

**General information**

| Characteristics | Modalities (fill before the interview) |
| --- | --- |
| Department |  |
| Town |  |
| Borough |  |
| Village |  |
| Hamlet or neighbourhood |  |

**Identification of the respondent**

1-Name :………………………………………………………………….

2-First name :………………………………………………………………………

3-Age :………… 4-Sex : male ` female

5-Ethnic group : bariba ;peuhl ;somba ;otamari ;natimba ;pila-pila lokpa ;yandé ;berba ;lama ;mahi ;nago Others……………………………………………………………..

6-Religion : muslim ; Christian ; Endogenous religion Atheist

Other religions (specify) …………………………….………………….

7- Education : No academic education Primary school

Secondary School University school

9-Profession :…………………………………………………………………………

1. How the *wagashi* dyeing is done ?

| Unit operations | Response | | Ingredient | Response | |
| --- | --- | --- | --- | --- | --- |
|  | Yes | No |  | Yes | No |
| *Wagashi* cooking |  |  | *Kanwu* |  |  |
|  |  |  | Bicarbonate |  |  |
|  |  |  | Salt |  |  |
| Colorant extraction  :  cool hot | | | *Kanwu* |  |  |
|  |  |  | Ash |  |  |
|  |  |  | Bicarbonate |  |  |

| Unit operations | Time allocated | Response | |
| --- | --- | --- | --- |
|  |  | Yes | No |
| *Wagashi*dyeing:  in cool watery extact  in warm watery extract  in hot watery extract during *wagashi* cooking | Less than 15 minutes |  |  |
|  | 15 minutes to 30 minutes |  |  |
|  | 30 minutes to 45 minutes |  |  |
|  | 45 minutes to 1 hour |  |  |
|  | 1 hour to 1 hour 30 minutes |  |  |
|  | 1 hour 30 minutes to 2 hours |  |  |
|  | Other time (specify) |  |  |

1. Does the intensity of the redness of the colorant help to reduce the time allocated to the dyeing?

Yes No

1. What are the reasons that motivate the *wagashi* dyeing ?

| Reasons | Yes | No |
| --- | --- | --- |
| Keep *wagashi* attractive |  |  |
| Hamper visual detection of spoiling of *wagashi* and standardise the colour |  |  |
| Delays the *wagashi* spoiling |  |  |
| Delay the fermentation of *wagashi* |  |  |
| Others : (specify) |  |  |

1. How do you appreciate the shelf-life of the following *wagashi* ?

|  | Indication of the shelf-life |
| --- | --- |
| Non-dyed *wagashi* bought from the Fulani | low moderate long |
| Non-dyed *wagashi* cooked with salt and *kanwu* | low moderate long |
| *Wagashi* cooked with salt, *kanwu* and dyed with sorghum biocolorant | low moderate long |

1. In slum times, what is the longest shelf-life have you experienced for dyed *wagashi*?

|  | Longest shelf-life |
| --- | --- |
|  |  |
| Dyed *wagashi* | 1 day 2 days 3 days  4 days 5 days 6 days  7 days 8 days 9 days  10 days 11 days 12 days  More than 12 days |

1. In slum times, what is the longest shelf-life have you experienced for non-dyed *wagashi*

|  | Longest shelf-life |
| --- | --- |
|  |  |
| Non-dyed *wagashi* | 1 day 2 days 3 days  4 days 5 days 6 days  7 days 8 days 9 days  10 days 11 days 12 days  More than 12 days |

1. In slum times, what is the frequency of a repetitive cooking and dyeing

|  | Frequency |
| --- | --- |
|  |  |
| Repetitive cooking with salt and dyeing | 1 day 2 days 3 days  4 days 5 days 6 days |
| Repetitive dyeing | 1 jour 2 days 3 days  4 days 5 days 6 days |

1. What size of *wagashi* do you sell ?

small medium large

1. Why do you choose to sell this (or these) size (s) of *wagashi*  ?

………………………………………………………………………………………………………………………………………………………………………………………………………………………………………………………………………………………………………

1. Is the quantity of leaf sheaths differ according to the size of *wagashi*?

Yes No

If Yes, explain :………………………….…………………………………………....................

…………………………………………………………………………………………………...

…………………………………………………………………………………………………...

…………………………………………………………………………………………………..

1. Does the time allocate to the dyeing depend on the size of the *wagashi* ? Yes No

If Yes, explain :…………………………………………………….............................................

…………………………………………………………………………………………………...

…………………………………………………………………………………………………...

…………………………………………………………………………………………………..

1. Could you provide information on the inputs for *wagashi* dyeing and their price ?

| Step | Inputs | Quantity | Price | Cost |
| --- | --- | --- | --- | --- |
| Cuisson du *wagashi* | *wagashi* |  |  |  |
|  | water |  |  |  |
|  | *kanwu* |  |  |  |
|  | Salt |  |  |  |
|  | Plastic bags |  |  |  |
|  | wood |  |  |  |
|  | Others (Specify) |  |  |  |
|  | Total 1 |  |  |  |
| *Wagashi* dyeing | Sorghum leaf sheaths |  |  |  |
|  | water |  |  |  |
|  | *kanwu* |  |  |  |
|  | Others (Specify) |  |  |  |
|  | Total 2 |  |  |  |

1. How much is dyed *wagashi* ?

………………………………………………………………………………………………..

1. How much is non-dyed *wagashi*?

………………………………………………………………………………………………..

1. How much time is allocated to the following tasks ?

| Tasks | Time allocated (minutes) |
| --- | --- |
| *Wagashi* cooking |  |
| *Wagashi* dyeing |  |
| Total |  |
